# Role of high-temperature requirement serine protease A 2 in rheumatoid inflammation

**DOI:** 10.1186/s13075-023-03081-z

**Published:** 2023-06-07

**Authors:** Gi Heon Jeong, Min-Kyung Nam, Wonhee Hur, Seolhee Heo, Saseong Lee, Eunbyeol Choi, Jae Hyung Park, Youngjae Park, Wan-Uk Kim, Hyangshuk Rhim, Seung-Ah Yoo

**Affiliations:** 1 Department of Biomedicine & Health Sciences, Department of Medical Life Sciences, College of Medicine, The Catholic University of Korea, Seoul, Korea; 2 Center for Integrative Rheumatoid Transcriptomics and Dynamics, The Catholic University of Korea, Seoul, Korea; 3 Division of Chronic Viral Diseases, Center for Emerging Virus Research, Korea National Institute of Health, Cheongju, Korea; 4 School of Chemical Engineering, College of Engineering, Sungkyunkwan University, Suwon, Korea; 5 Department of Internal Medicine, The Catholic University of Korea, Seoul, Korea

**Keywords:** Rheumatoid arthritis, Fibroblast-like synoviocytes, High-temperature requirement serine protease A, Inflammation

## Abstract

**Background:**

High-temperature requirement serine protease A 2 (HtrA2) is known to be involved in growth, unfolded protein response to stress, apoptosis, and autophagy. However, whether HtrA2 controls inflammation and immune response remains elusive.

**Methods:**

Expression of HtrA2 in the synovial tissue of patients was examined using immunohistochemistry and immunofluorescence staining. Enzyme-linked immunosorbent assay was used to determine the concentrations of HtrA2, interleukin-6 (IL-6), interleukin-8 (IL-8), chemokine (C-C motif) ligand 2 (CCL2), and tumor necrosis factor α (TNFα). Synoviocyte survival was assessed by MTT assay. For the downregulation of HtrA2 transcripts, cells were transfected with HtrA2 siRNA.

**Results:**

We found that the concentration of HtrA2 was elevated in rheumatoid arthritis (RA) synovial fluid (SF) than in osteoarthritis (OA) SF, and its concentrations were correlated with the number of immune cells in the RA SF. Interestingly, HtrA2 levels in the SF of RA patients were elevated in proportion to synovitis severity and correlated with the expression of proinflammation cytokines and chemokines, such as IL-6, IL-8, and CCL2. In addition, HtrA2 was highly expressed in RA synovium and primary synoviocytes. RA synoviocytes released HtrA2 when stimulated with ER stress inducers. Knockdown of HtrA2 inhibited the IL1β-, TNFα-, and LPS-induced release of proinflammatory cytokines and chemokines by RA synoviocytes.

**Conclusion:**

HtrA2 is a novel inflammatory mediator and a potential target for the development of an anti-inflammation therapy for RA.

**Supplementary Information:**

The online version contains supplementary material available at 10.1186/s13075-023-03081-z.

## Background

Rheumatoid arthritis (RA) is a chronic inflammatory condition that can cause joint degeneration over time [1]. In RA joints, a variety of cell types, including innate immune cells, adaptive immune cells, endothelial cells, and fibroblast-like synoviocytes (FLSs), are activated. In particular, synoviocytes play an important role in the invasive pannus and directly contribute to chronic inflammation and cartilage degradation [1, 2]. They are capable of producing matrix-degrading enzymes and cytokines, such as IL-6 and IL-8, as well as angiogenic factors. Furthermore, fibroblast-like synoviocytes from inflamed joints can grow abnormally, migrate to the local environment, and exhibit tumor-like features [3, 4].

Synovial tissue inflammation is known to contribute to RA development. It exists in all stages of RA, even in its early stages. Numerous proinflammatory cytokines, including tumor necrosis factor (TNF), IL-6, IL-8, IL-17, and CCL2, contribute to the progression of RA [5]. An increase in joint infiltration by immune cells, specifically macrophages, has been closely linked to elevated CCL2 levels in RA [6]. Synovial inflammation also promotes synovial angiogenesis in the synovium, which, in turn, accelerates inflammation [3, 7]. Therefore, controlling the inflammatory response is a promising strategy for the treatment of RA.

Human high-temperature requirement serine protease A (HtrA2), also known as Omi, is a mitochondrial serine protease. It is an apoptosis-inducing protein that is released from the mitochondrial intermembrane space into the cytosol following an apoptotic stimulus [8]. HtrA2 plays a role in the progression of neurodegenerative diseases, prostate cancer, and hepatocellular carcinoma [8, 9]. HtrA1, a paralog of HtrA2, is also involved in the development of various skeletal diseases including RA [10, 11]. Of note, HtrA1 and HtrA2 of the HtrA2 family of serine proteases appear to share substrate specificity. These studies suggest that HtrA2 modulates the progression of RA [10, 12]; however, the molecular mechanisms through which HtrA2 controls inflammation and the immune response are unclear. Moreover, most studies on its disease association have been carried out using the proapoptotic mitochondrial serine protease. In the present study, we demonstrate that HtrA2 mediates the pathogenesis of inflammatory arthritis. HtrA2 was observed to be abundant in RA synovium samples and synovial fluids from patients. Synovial HtrA2 levels were directly associated with synovial IL-6, IL-8, and CCL2 levels (representative proinflammatory cytokines/chemokines). In addition, ER stress stimuli such as tunicamycin and thapsigargin released HtrA2 in cultured synoviocytes. For the first time, we demonstrated that HtrA2 is secreted extracellularly during ER stress-induced apoptosis. Furthermore, siRNA-mediated downregulation of HtrA2 transcripts reduced the production of inflammatory cytokines and chemokines (IL-6, IL-8, and CCL2) in RA FLSs while eradicating IL1β-, TNFα-, or LPS-triggered cytokine production. Overall, our data show that HtrA2 is a novel inflammatory mediator. Collectively, our finding provides new insights into the use of HtrA2 as a novel biomarker of RA and the design of next-generation therapeutic strategies for RA, as the production of other inflammatory mediators is reduced by modulating the amount of HtrA2.

## Methods

### Study population and sampling of SF and serum

Individuals with RA who met the 2010 ACR/EULAR categorization criteria [13] and osteoarthritis (OA) controls were enrolled between August 2015 and December 2018. SF samples were collected from individuals with RA (*n* = 72) and an OA control group (*n* = 61), who were subjected to arthrocentesis for swollen joints. SFs were centrifuged at 6000*g* for 15 min after they were aspirated from affected joints. Supernatants were stored at −80 °C.

### Assessment of synovitis severity by ultrasonography

Sonographic evaluations of joints including grayscale US (GSUS) and power Doppler US (PDUS) were conducted as previously reported [14]. In summary, GSUS defined the degree of synovial hypertrophy as follows: grade 0 = no synovial hypertrophy; grade 1 = minimal synovial hypertrophy; grade 2 = moderate synovial hypertrophy; and grade 3 = severe synovial hypertrophy. PDUS scores were evaluated by the degree of vascularity within synovium of joints as follows: grade 0 = absence of Doppler signal; grade 1 = minimal Doppler signal; grade 2 = moderate Doppler signal (≥grade 1 but < 50% of Doppler signals in the total background); grade 3 = high Doppler signal (> 50% of Doppler signals in the total background). Individuals were characterized as having active synovitis if GSUS grade or more ≥2 or if PDUS grade was 1 or more ≥1.

### Isolation and culture of fibroblast-like synoviocytes (FLS)

Fibroblast-like synoviocytes (FLSs) were isolated from synovial tissues of individuals with rheumatoid arthritis (RA) and osteoarthritis (OA) who had complete joint replacement surgery. FLSs were extracted from synovial tissues as previously reported [15, 16]. FLSs from passages 4–8 were seeded into 24-well plates at a density of 2×10^4^ cells/well in Dulbecco’s modified Eagle’s medium (DMEM) supplemented with 10% Fetal bovine serum (FBS), penicillin, streptomycin, and amphotericin B.

### Downregulation of HtrA2 transcripts

To downregulate HtrA2 transcripts, Lipofectamine 3000 (Invitrogen) was used to transfect RA FLSs with HtrA2 siRNA (Santa Cruz Biotechnology, Inc.). Control siRNAs were also obtained from Santa Cruz Biotechnology, Inc. HtrA2 mRNA and protein expression levels in FLSs were evaluated by RT-PCR including Western blot analysis relying on anti-HtrA2 monoclonal antibody (Abcam), respectively. To detect HtrA2 mRNA, RT-PCR was conducted with the following HtrA2-specific primers: sense, 5′-GCACTGCAGAACACGATCAC-3; and antisense, 5′-GGGACCTCCAGAGTTTCCAA-3′. GAPDH mRNA expression was used as an internal control.

### Enzyme-linked immunosorbent assay (ELISA)

ELISA kits for human IL-6 (R&D Systems), IL-8 (R&D Systems), CCL2 (R&D Systems), TNFα (R&D Systems), and HtrA2 (R&D System) were used to measure cytokine concentration in culture supernatants and SFs.

### Cell viability: MTT assay

Synoviocyte viability was assessed by an MTT assay as previously reported [15, 16].

### Immunohistochemical staining

Formalin-fixed paraffin-embedded synovial tissue specimens sectioned at 5 μm in thickness were obtained from four RA patients and four OA patients. The tissue was deparaffinized in xylene, rehydrated in a gradient series of ethanol, microwaved, and treated with 3% hydrogen peroxide to inhibit endogenous peroxidase activity. Following blocking nonspecific binding using 10% normal horse serum for 1 h at ambient temperature, the slides were treated with rabbit anti-HtrA2 Ab (1:100; Abcam) overnight at 4°C. Isotype control antibody was applied as a negative control. After washing, the slides were incubated for 30 min at room temperature with goat anti-rabbit IgG secondary Ab (ImmPRESS HRP^TM^ REAGENT KIT; Vector Laboratories). 3,3′-Diaminobenzidine tetrahydrochloride was used to detect positive cells.

### Immunofluorescence staining

A 4% paraformaldehyde solution was used to fix RA-FLSs for 20 min, then permeabilized using 0.1% Triton X-100/PBS for 15 min at ambient temperature. Following blocking for 1 h with a 10% solution of normal donkey serum, slides were stained with Abs to HtrA2 (1:100; Abcam) and Tom20 (1:100; Santa Cruz Biotechnology) for 2 h at ambient temperature. Each slide was washed three times with PBS, then incubated with an Alexa Fluor 488-tagged anti-rabbit IgG Ab (1:1000; Abcam) or Alexa Fluor 594-tagged anti-mouse IgG Ab (1:1000; Abcam) for 2 h at ambient temperature. DAPI was used to stain the nuclei and the glass slides were mounted with Vectashield Antifade Mounting Medium (Vector laboratories). CellLight Reagents BacMam 2.0 was used to stain intracellular organelles such as the mitochondria, and lysosomes, according to the manufacturer’s instructions. A confocal microscope was used to examine the stained cells (LSM 800; Cari Zeiss, Germany). The raw and deconvolved images were analyzed quantitatively using Imaris x64 v7.2 (Oxford instruments) and Zeiss ZEN software.

### Western blot analysis

After lysing RA-FLS and OA-FLS samples in lysis buffer, insoluble material was removed by centrifugation for 20 min (14,000 rpm at 4°C). The BCA protein assay (Thermo Fisher Scientific) was used to estimate the protein concentration. SDS-PAGE was used to separate the proteins, which were then transferred to polyvinylidene fluoride membranes. HtrA2 (1:2000; Abcam) and the membranes were treated with β-actin antibodies (1:1000; Santa Cruz Biotechnology). After washing and incubation with secondary antibody, an enhanced chemiluminescent approach was used to visualize the protein bands.

### Statistical analysis

The data are presented as the mean ± standard error of the mean (s.e.m.). The Mann-Whitney *U* test, Wilcoxon matched pairs test, and one-way (or two-way) ANOVA test were applied. The Spearman correlation test was used to examine the relationships between cytokines and HtrA2 levels in RA SFs. For statistical evaluations, paired or unpaired *t*-test was utilized, as specified in each group. *P*-values less than 0.05 were considered statistically significant. GraphPad Prism software v9 was used for all statistical analyses.

## Results

### HtrA2 levels in the SF of patients with RA correlated with disease activity and inflammatory cytokine levels

**Fig. 1:**
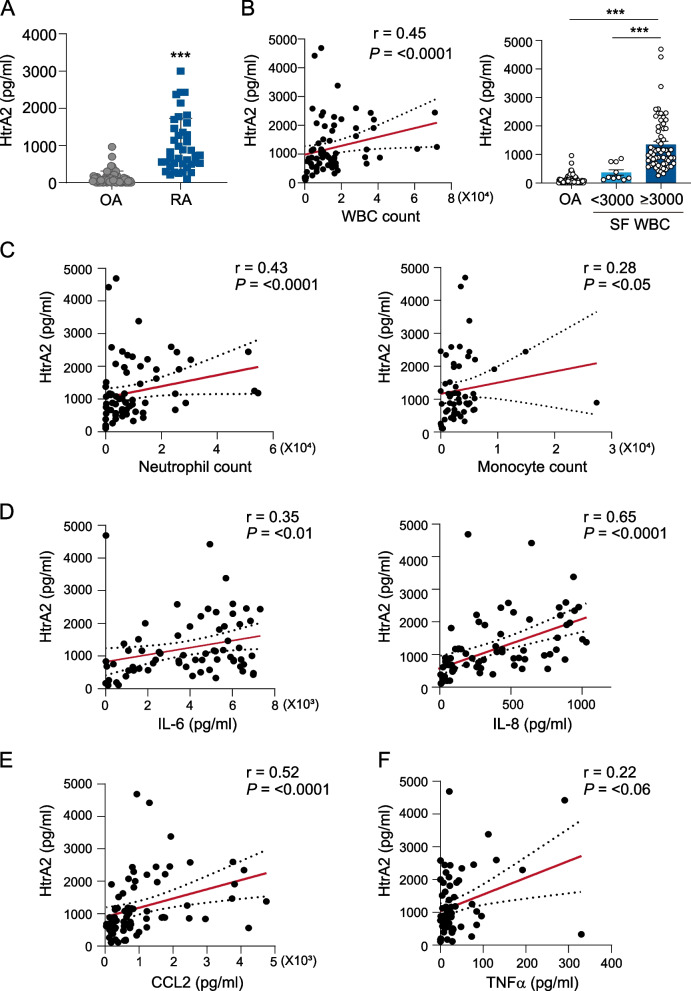
Correlation between HtrA2 and inflammatory cytokine levels. **A** HtrA2 concentrations in synovial fluids (SFs) of individuals with RA (*n*=72) or OA (*n*=61). **B**, **C** Fluid concentrations of HtrA2 depending on the count of white blood cells (WBCs, *p* < 0.0001), neutrophils (*p* < 0.0001), or monocytes (*p* < 0.05). **D**, **E** Correlation of HtrA2 levels with IL-6 (*p* < 0.01), IL-8 (*p* < 0.0001), CCL2 (*p* < 0.0001), and TNFα (*p* < 0.06) levels in the SF of patients with RA. Data present the mean ± s.e.m. ***p < 0.001. *P* values were determined by Mann-Whitney U test (**A**), Spearman correlation (**C**–**F** and left panel of **B**), or one-way ANOVA (right panel of **B**)

**Fig. 2:**
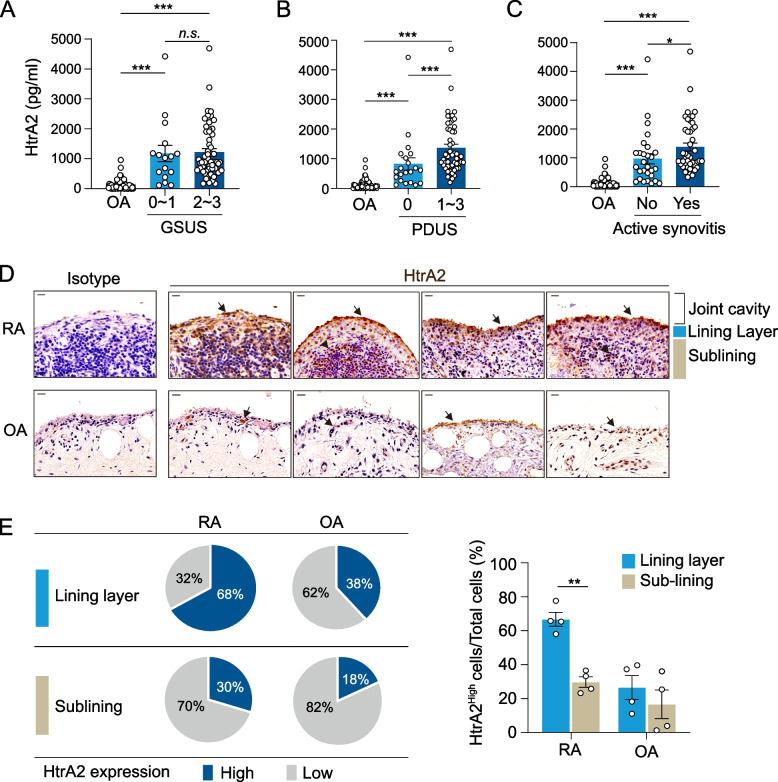
Expression of HtrA2 in RA synovial fluids and the synovium. **A** HtrA2 levels in the SF of RA patients with considerable synovial hypertrophy (grayscale US, GSUS 2 and 3) compared with those with little or mild synovial hypertrophy (GSUS 0 and 1). **B** HtrA2 concentrations in RA patients with and without enhanced vascularity (power-Doppler US, PDUS 1–3). (PDUS 0). **C** HtrA2 concentrations in RA patients with and without active synovitis. Active synovitis was classified as GSUS ≥2 or PDUS ≥1. **D** Anti-HtrA2 antibody or isotype control antibody immunohistochemical staining of rheumatoid arthritis (RA, *n*=4) and osteoarthritis (OA, *n*=4) synovium. HtrA2 positivity was found in the cell lining layer (arrow), as well as in infiltrating leukocytes in the sublining layer (arrowhead). Scale bar, 20 μm.** E** Summary of the percentage of cells in each layer. Data are the mean ± s.e.m. *p < 0.05, **p < 0.01, ***p < 0.001. *P* values were determined by one-way ANOVA test (**A**–**C**) or two-way ANOVA (**E**)

We first compared HtrA2 levels in SF between patients with RA and OA. As demonstrated in Fig. 1A, the concentration of HtrA2 in RA SF (*n*=72) was 8.7-fold higher compared with that in OA SF (*n*=61). To assess the clinical importance, we examined the relationship between synovial HtrA2 and known RA disease markers. HtrA2 concentrations in SF were significantly correlated with WBC (*r* = 0.45, *p* < 0.0001), neutrophil (*r* = 0.43, *p* < 0.0001), and monocyte (*r* = 0.28, *p* < 0.05) (Fig. 1B, C). Interestingly, synovial HtrA2 levels were moderately associated with synovial IL-6, IL-8, and CCL2 levels, which are representative proinflammatory cytokines and chemokines (Fig. 1D, E), despite having weak associations with SF TNFα levels (Fig. 1F). Overall, our clinical findings point to a connection between synovial HtrA2 and inflammatory responses in RA joints. We then wondered if HtrA2 could indicate synovitis severity as evaluated by US in RA patients. The findings demonstrated that HtrA2 concentrations did not alter significantly depending on the presence of severe synovial hypertrophy on GSUS (Fig. 2A). HtrA2 concentrations on PDUS were higher in patients with enhanced vascularity (grades 1–3) than in those without (grade 0) (Fig. 2B). When participants were separated into two groups based on the presence of active synovitis (GSUS ≥2 or PDUS ≥1), HtrA2 levels were considerably higher in patients with active synovitis compared with those with inactive synovitis (Fig. 2C). In RA patients, these levels may indicate the degree of synovitis as well as local and systemic inflammatory problems.

### Expression of HtrA2 in RA synovium

To determine the distribution and localization of HtrA2 in synovial tissue, we stained the synovial membranes of four RA patients and four OA patients using immunohistochemistry (Fig. 2D). HtrA2 expression was high in all RA synovial tissue slides. The lining layer and infiltrating cells of the RA synovial membranes were stained positively. HtrA2 expression was also detected in the OA synovium, although the density was weak. The distribution of cells with or without an HtrA2-positive signal was determined by dividing the lining layer and sublining layer. The percentage of HtrA2-positive cells in the lining layer was 68% in RA patients, compared with 38% in the OA patients (Fig. 2E). The proportion of HtrA2-positive cells in the lining layer was increased in RA compared with OA, whereas it was lower in the sublining layer of the RA samples. Immunofluorescence staining revealed that HtrA2-expressing cells in RA synovium were positive for CD55, indicating lining layer. However, CD90-positive cells, which indicate the sublining layer, were not colocalized with HtrA2-expressing cells (Supplementary Fig. 1). These results indicate that cells expressing HtrA2 are more concentrated in the lining layer in RA compared with that in OA.

### HtrA2 is expressed in primary synoviocytes and upregulated by proinflammatory stimuli

**Fig. 3:**
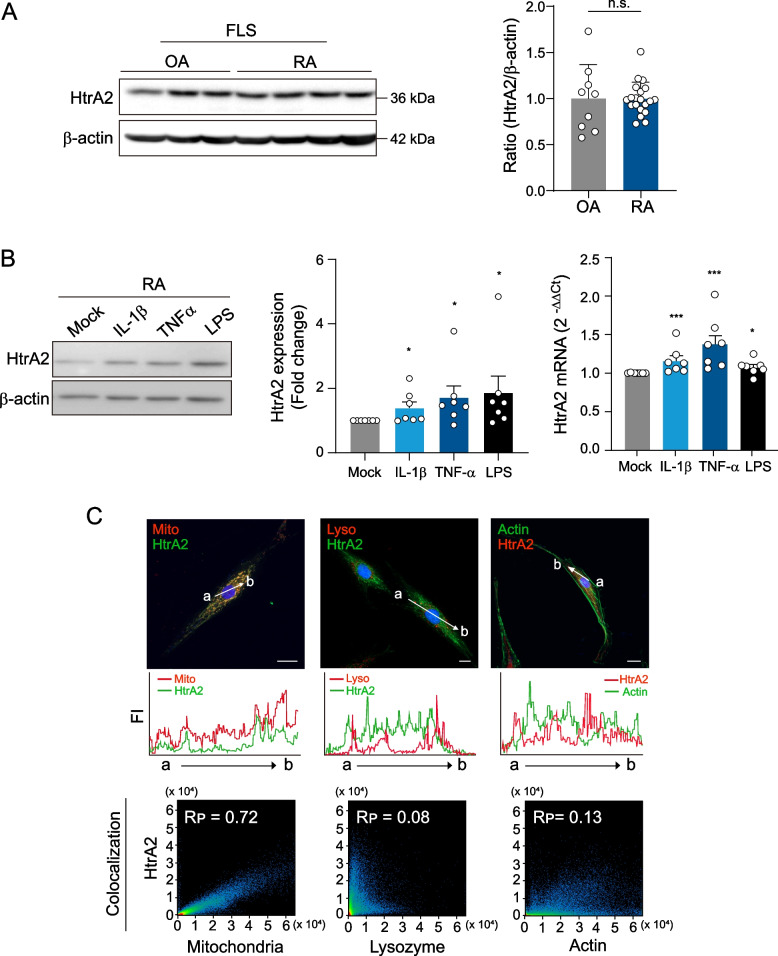
Increase in HtrA2 expression in FLSs by proinflammatory stimuli. **A** HtrA2 protein expression in RA-FLSs (*n*=20) and OA-FLSs (*n*=9) was assessed by western blot analysis. **B** RA-FLSs were stimulated with TNFα (10 ng/ml), IL1β (1 ng/ml), or LPS (1 μg/ml) in 1% FBS DMEM for 6 h or 12h. The mRNA and protein expression levels of HtrA2 in synoviocytes were determined by western blot analysis (left panel, *n*=5) and RT-PCR (right panel, *n*=7). Data are the mean ± s.e.m. *p < 0.05, **p < 0.01, ***p < 0.001. *P* values were determined by one-way ANOVA test. **C** Double immunofluorescence staining of synoviocytes. Cells were stained with Ab for HtrA2, mitochondria marker (RFP), F-actin marker (GFP), and lysosome marker (RFP) in RA-FLSs. Nuclei were stained with DAPI (blue). The fluorescence signal intensity (FI) was quantified along the white arrow. Scale bar, 20 μm. The colocalization study was carried out utilizing raw and deconvolved images, as well as Imaris, and ZEN software. The Rp indicates Pearson’s colocalization coefficient

The lining layer of the synovium is composed of synovial cells, such as macrophage-like synoviocytes, and FLS. FLSs are the most abundant cell type in the inflamed RA synovial membrane. We compared HtrA2 baseline expression levels in cultured FLSs from RA and OA patients. The HtrA2 protein was found in twenty RA FLSs and nine OA FLSs with identical passage numbers, according to Western blot analysis (Fig. 3A). RA FLSs are exposed to inflammatory cytokines such as IL1β and TNFα in swollen joints [3, 4]. Therefore, we determined the effects of inflammatory cytokines on HtrA2 expression in cultured FLS of the RA cases. As shown in Fig. 3B, HtrA2 expression levels were increased following treatment with IL1β, TNFα, or LPS in cultured RA FLSs. These results suggest that HtrA2 is expressed in the synovium of RA cases by proinflammatory stimuli. HtrA2 is mainly located in the mitochondria to control mitochondrial homeostasis in mice and human [8]. To determine the localization and distribution of HtrA2 in primary synoviocytes, we performed immunocytochemical staining using an anti-HtrA2 Ab. As expected, HtrA2 was primarily localized in the mitochondria of synoviocyte (Fig. 3C).

### Release of HtrA2 into the extracellular space

**Fig. 4:**
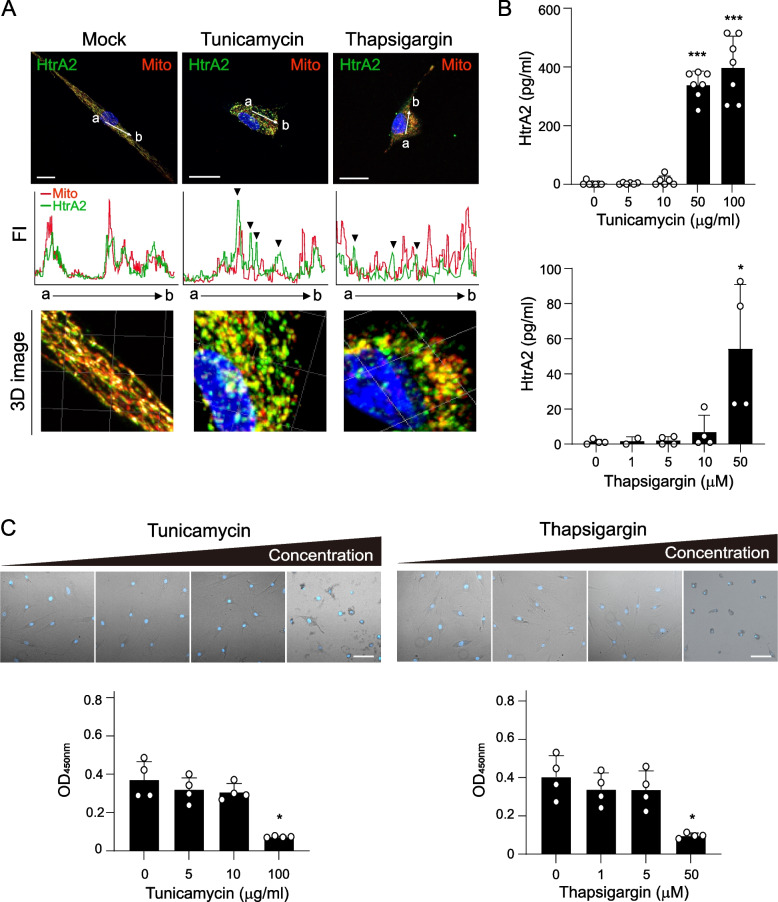
The release of HtrA2 into the extracellular space. **A** Tunicamycin and thapsigargin induce HtrA2 release from the mitochondria into the cytosol. RA-FLSs were treated with 20 μg/ml tunicamycin or 20 μM thapsigargin for 16 h, after immunostaining with HtrA2 (green) and Tom20 (Mito, red). The fluorescence signal intensity (FI) was quantified along the white arrow. Scale bar, 20 μm. 3D images prepared with Imaris software. **B** HtrA2 expression was significantly elevated in the supernatant of RA-FLSs following ER stress-triggered apoptosis. RA FLSs (2×10^4^ cells) were cultured for 24h with 1% FBS DMEM in the presence of tunicamycin (*n*=7) or thapsigargin (*n*=4). Concentrations of HtrA2 were determined by ELISA. **C** FLSs treated with 100 μg/ml tunicamycin or 50 μM thapsigargin became spherical, shrunken, and detached from the bottom of the culture plates under phase-contrast microscopy, whereas untreated cells remained bipolar (upper panel). The nuclei were stained with DAPI (blue). Cell viability was determined by MTT assay (lower panel, *n*=4). Data presented as mean ± s.e.m. *p < 0.05, ***, *p* < 0.001. *P* values were determined by one-way ANOVA test (**B** and **C**)

Next, we examined the potential mechanism by which HtrA2 is released into the RA synovial cavity as shown in Fig. 1. HtrA2 is an apoptosis-triggering protein that is discharged from the mitochondria into the cytosol in response to an apoptotic signal [17, 18]. A recent study showed that HtrA2 expression is detectable in the extracellular space of human umbilical vein endothelial cells during apoptosis [19]. To determine whether HtrA2 is released by primary synoviocytes, we stimulated FLSs with tunicamycin or thapsigargin as an ER stress inducer. Interestingly, after this stimulation, HtrA2 was redistributed to the cytoplasm from the mitochondria (Fig. 4A). When RA FLSs were stimulated with 100 μg/ml tunicamycin, HtrA2 concentration was increased by up to 400-fold compared to its spontaneously produced level (Fig. 4B). HtrA2 release was also induced by treatment with 50 μM thapsigargin, which stimulated cell death conditions (Fig. 4B, C). These results indicate that HtrA2 is released by synoviocytes upon ER-stress-induced apoptosis, which suggests that this pathway provides extracellular HtrA2 observed in RA SFs.

### Reduction of inflammatory cytokines by downregulation of HtrA2

**Fig. 5:**
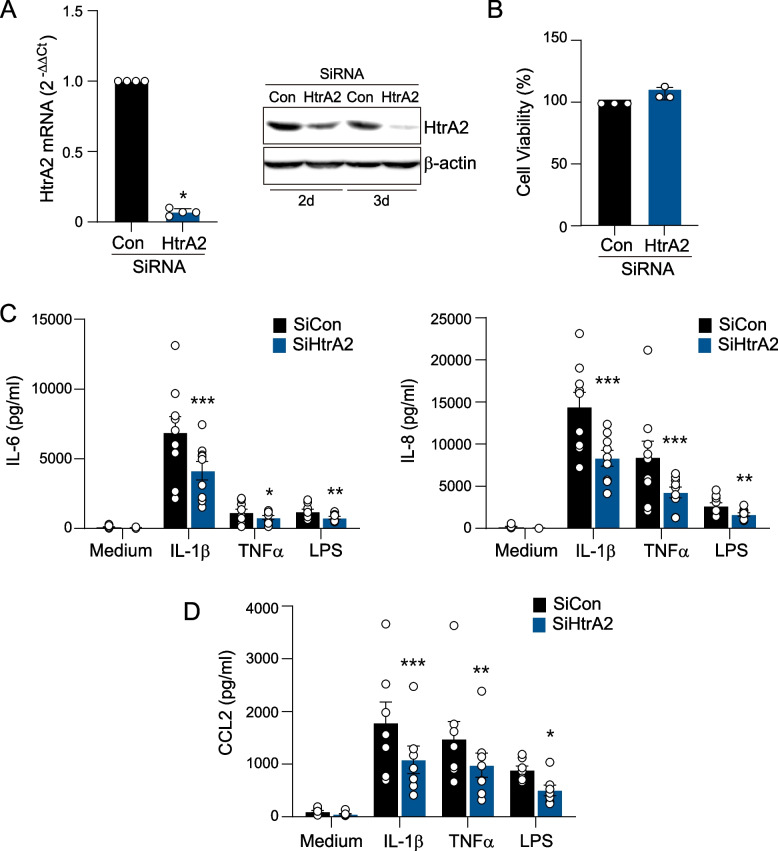
The effect of HtrA2 siRNA on cytokine production.** A** Transfection efficiency of HtrA2 siRNA was measured by RT-qPCR (left panel, *n*=4) including western blotting (right panel). Twelve hours after transfection with siRNA for HtrA2, mRNA expression levels were determined by RT-qPCR analysis. **B** Fifty-eight hours after transfection with siRNA for HtrA2, cell viability was measured by an MTT assay (*n*=3). **C** Effect of HtrA2 knockdown on IL1β-, TNFα-, or LPS-induced increases of IL-6 and IL-8 production in RA synoviocytes. At 24 h after HtrA2 siRNA transfection, RA FLSs (*n*=9) were treated with IL1β (1 ng/ml), TNFα (10 ng/ml), or LPS (1 μg/ml) in 1% FBS DMEM for 24 h. Cytokine production was quantified by ELISA. **D** ELISA revealed CCL2 production by synoviocytes (*n*=7). Data represent the mean ± s.e.m. *p < 0.05, **p < 0.01, ***p < 0.001 versus siCon treated cells. *P* values were determined by Mann-Whitney *U* test (**A**) or Two-way ANOVA test (**C**, **D**)

FLSs of patients with RA can produce cytokines and/or chemokines, such as IL-8, IL-6, and CCL2 [3, 4]. Therefore, we examined whether HtrA2 mediates cytokine production in synoviocytes using HtrA2 siRNA transfection. As shown in Fig. 5A, we first confirmed HtrA2 knockdown in RA FLSs. The results of real time-PCR and immunoblot analysis suggested that HtrA2 expression was significantly lower in HtrA2 transfected cells. Stimulating FLSs with IL1β, TNFα, or LPS for 1 day generally increases the levels of inflammatory cytokines in RA FLSs. Knockdown of HtrA2 inhibited the production of IL1β-, TNFα-, or LPS-stimulated proinflammatory cytokines (IL-6 and IL-8) by RA FLSs without causing cell death, as determined by the MTT viability assay (Fig. 5B, C and Supplementary Fig. 2). Consistently, CCL2 was significantly reduced in the presence of HtrA2 siRNA (Fig. 5D). In OA FLS, decreased cytokine expression following HtrA2 knockdown was weaker compared with that in RA FLSs (Supplementary Fig. 3). These findings suggest that HtrA2 directly regulates the production of inflammatory cytokines and chemokines in RA FLSs.

## Discussion

HtrA2 is localized to the mitochondrial intermembrane space and influences the physiology of mitochondrial homeostasis [8, 9, 20]. Its aberrant expression has been linked to a variety of illnesses, including ovarian cancer, endometrial cancer, and neurological disorders [21–24]. In the present study, we demonstrated that HtrA2 is expressed in RA synovium and its expression is increased in synoviocytes during proinflammatory conditions. HtrA2 levels were markedly higher in the fluids of RA patients compared with that in the OA controls. To determine the relationship between HtrA2 expression and the severity of RA further, we examined its correlations with clinical variables in RA patients.

Previous research has found that HtrA2 contributes to the pathogenesis of autoimmune arthritis by regulating Th17 cells [7]. These studies demonstrate that HtrA2 can regulate RA in part through the Th17 cell differentiation of STAT3 [7]. In one study, UCF-101 (5-[5-(2-nitrophenyl) furfuryl iodine]-1,3-diphenyl-2-thiobarbituric acid), which is an inhibitor of HtrA2, seems to relieve pulmonary inflammation by reducing the generation of inflammatory cytokines [25]. HtrA2 deficiency has recently been shown to reduce the production of proinflammatory cytokines in BMDMs triggered by LPS or CpG, which suggests that HtrA2 is an immune response regulator [26]. In the present study, we clearly demonstrated that HtrA2 was a critical regulator of the production of inflammatory cytokines through HtrA2 knockdown. In particular, IL1β-, TNFα-, or LPS-induced increases of IL-6, IL-8, and CCL2 were completely blocked by HtrA2 siRNA. RA-FLSs actively participate in chronic inflammation by secreting inflammatory cytokines including IL-8 and IL-6 [3, 4]. Furthermore, CCL2 levels were significantly upregulated in RA patients [6, 27]. In the RA synovium, activated synoviocytes are responsible for the synthesis of CCL2, which exacerbates and sustains inflammation by recruiting inflammatory immune cells, primarily macrophages and monocytes [6, 28–30]. In this regard, anti-inflammatory agents targeting RA-FLSs may exhibit a therapeutic benefit. The present study showed that HtrA2 expression was associated with increases of proinflammatory cytokine production in synoviocytes.

We observed the release of HtrA2 from mitochondria into the culture medium following treatment with an ER stress inducer. Several scientists have postulated that during apoptosis, cytochrome c is released from the mitochondria into the cytosol and ultimately into the extracellular space [31–33]; however, the release of HtrA2 into the extracellular space during apoptosis has yet to be established [19]. In the present study, we demonstrated that HtrA2 release from RA FLSs into the extracellular environment was implicated in ER-stress-induced apoptosis. Previous studies have indicated that RA joints are subject to ER stress and that ER stress-related gene signatures are expressed in RA synovial cells [15, 16]. We hypothesize that chronic exposure of FLSs to ER stress within joints induces apoptosis, thereby releasing HtrA2.

HtrA2 is a potential biomarker for mitochondrial-induced apoptosis in the joint. However, its therapeutic utility is speculative, and further research is needed to fully grasp its role. Furthermore, there is no evidence that HtrA2 is an apoptotic marker; it might just be a marker of severe necrosis and cell lysis. Finally, our findings should be validated by larger patient population studies including further exploration into the physiology of HtrA2. More studies are necessary to determine whether HtrA2 can attach to a specific receptor or transport system at the plasma membrane and whether extracellular HtrA2 activates proapoptotic signaling pathways.

Overall, we demonstrated that HtrA2, beyond its classical role as a serine protease in the mitochondria, can acquire a new function as an inflammation regulator, exhibiting functional diversity with mitochondrial proteins. Our findings provide insight into the pathogenic mechanism of mitochondrial protein and highlight the significance of HtrA2 as a possible therapeutic target, as it is a frequent trigger of the inflammatory process.

## Conclusion

In conclusion, increased HtrA2 levels in SFs are associated with RA, which may be related to inflammatory cytokine levels. Our findings suggest that synovial HtrA2 could be a new biomarker for the diagnosis of RA. Furthermore, HtrA2 may be an effective RA treatment target capable of reducing inflammation.

## Supplementary Information

**Additional file 1: Supplementary figure 1.** Double immunofluorescence staining of HtrA2 and CD55 in synovial tissues of RA patient. **Supplementary figure 2.** The effect of HtrA2 siRNA on cell viability. **Supplementary figure 3.** The effect of HtrA2 siRNA on cytokine production in OA FLSs.

## Data Availability

The datasets used and/or analyzed during the current study are available from the corresponding author upon reasonable request.

## Abbreviations

BMDM: Bone marrow-derived macrophage
CCL2: Chemokine (C-C motif) ligand 2
ER: Endoplasmic reticulum
FLS: Fibroblast-like synoviocytes
GSUS: Grayscale ultrasonography
HtrA2: High-temperature requirement serine protease A 2
IL: Interleukin
LPS: Lipopolysaccharide
MTT: 3-(4,5-Dimethylthiazol-2-yl)-2,5-Diphenyltetrazolium Bromide
OA: Osteoarthritis
PDUS: Power Doppler ultrasonography
RA: Rheumatoid arthritis
SF: Synovial fluid
TNF: Tumor necrosis factor
Th: T helper
TOM20: Translocase of outer membrane 20
