# Supplementary material for: Role of high-temperature requirement serine protease A 2 in rheumatoid inflammation
Source: Arthritis Res Ther. 2023 Jun 7;25:96. doi: 10.1186/s13075-023-03081-z (PMC10246393; doi:10.1186/s13075-023-03081-z)
Supplement: Supplementary file 1 — Additional file 1: Supplementary figure 1. Double immunofluorescence staining of HtrA2 and CD55 in synovial tissues of RA patient. Supplementary figure 2. The effect of HtrA2 siRNA on cell viability. Supplementary figure 3. The effect of HtrA2 siRNA on cytokine production in OA FLSs. [file 13075_2023_3081_MOESM1_ESM.docx]

**Supplementary information**

**Role of High-temperature Requirement Serine Protease A 2 in Rheumatoid Inflammation**

Gi Heon Jeong ^1, 2, †^, Min-Kyung Nam ^1, †^, Wonhee Hur ^3^, Seolhee Heo ^1, 2^, Saseong Lee ^1, 2^, Eunbyeol Choi ^1, 2^, Jae Hyung Park ^4^, Youngjae Park ^5^, Wan-Uk Kim ^1, 2, 5,^ *, Hyangshuk Rhim ^1,^ *, and Seung-Ah Yoo ^1, 2,^ *

**Supplementary figure 1. Double immunofluorescence staining of HtrA2 and CD55 in synovial tissues of RA patient.** Sections were subsequently immunostained with Alexa Fluor 488-conjugated anti-rabbit IgG (green) for HtrA2 Ab and Alexa Fluor 594–conjugated anti-mouse IgG (red) for CD55 Ab (or CD90 Ab). Colocalization of HtrA2 (green) and CD55 (red) is visualized in yellow on the merged images. Nuclei were stained with DAPI (blue).


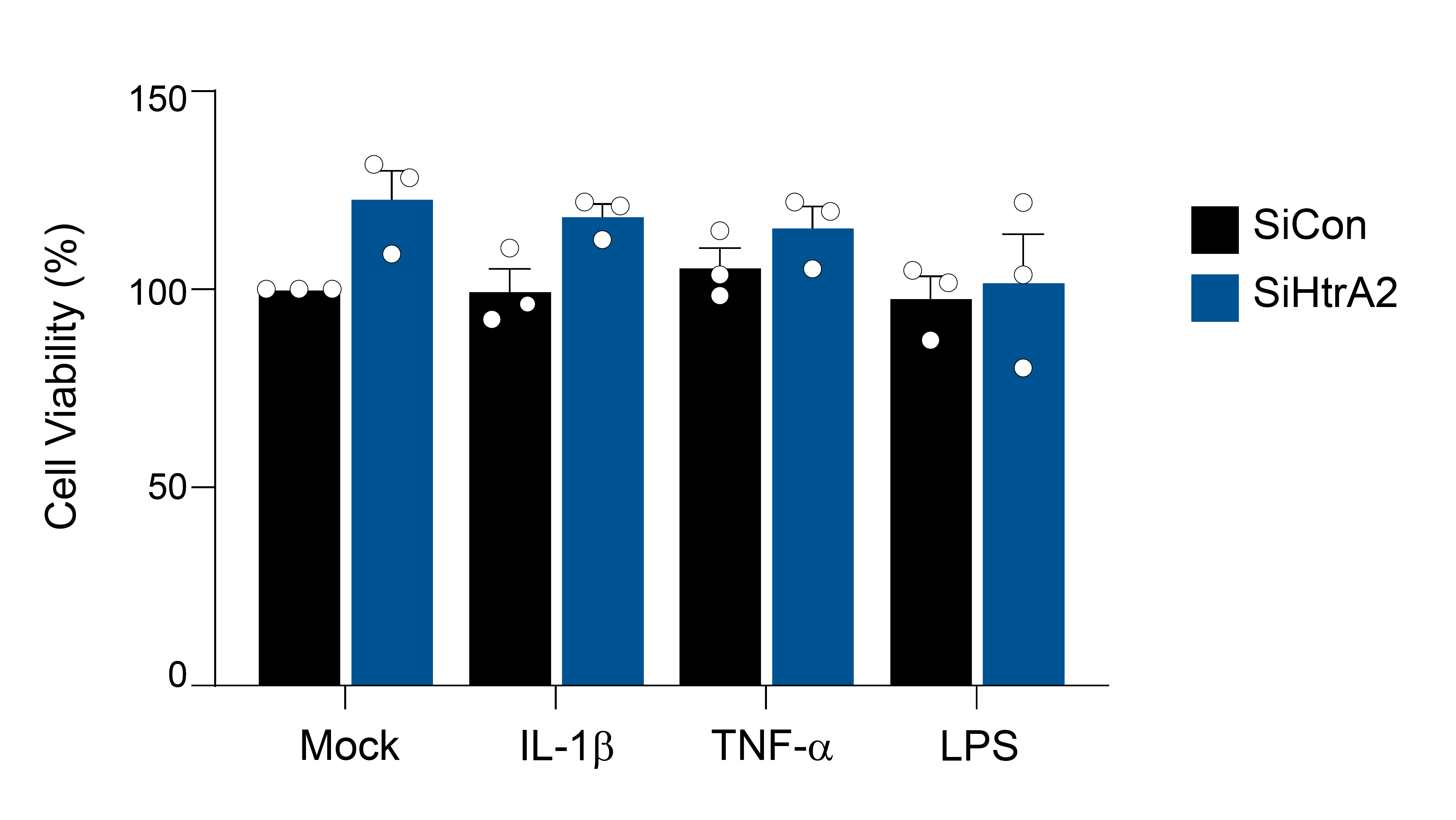


**Supplementary figure 2. The effect of HtrA2 siRNA on cell viability.** Cell viability was measured by an MTT assay (n=3).

**Supplementary figure 3. The effect of HtrA2 siRNA on cytokine production in OA FLSs. (A)** Transfection efficiency of HtrA2 siRNA was measured by RT-qPCR (n=4). **(B)** Cell viability was measured by an MTT assay (n=3). Effect of HtrA2 knockdown on IL1β-, TNFα-, or LPS-induced increases of IL-6 **(C)**, IL-8 **(D)**, and CCL2 **(E)** production in OA synoviocytes. At 24 h after HtrA2 siRNA transfection, OA FLSs (n=3) were treated with IL1β (1 ng/ml), TNFα (10 ng/ml), or LPS (1 μg/ml) in 1% FBS DMEM for 24 h. Cytokine production was quantified by ELISA.
